# Factors Associated With Needle Stick Injuries Among Healthcare Workers: A Retrospective Study in a Tertiary Care Hospital of Eastern India

**DOI:** 10.7759/cureus.72066

**Published:** 2024-10-21

**Authors:** Nipa Singh, Ipsa Mohapatra, Dipti Pattnaik, Subhra Snigdha Panda, Rashmita Pradhan, Kalpana Mund, Preety Mishra, Soumya Nayak, Gaurav Verma, Subham ravi Nayak, Adyasha Jena

**Affiliations:** 1 Medical Microbiology, Kalinga Institute of Medical Sciences, Bhubaneswar, IND; 2 Community Medicine, Kalinga Institute of Medical Sciences, Bhubaneswar, IND; 3 Pharmacology, SCB Medical College, Cuttack, IND; 4 Medicine, Kalinga Institute of Medical Sciences, Bhubaneswar, IND

**Keywords:** healthcare workers, needle stick injuries, occupational hazard, risk factors, serological status

## Abstract

Background: Needle stick injury (NSI) is one of the significant and serious health hazards encountered by healthcare workers (HCWs), as it is a risk factor for transmission of blood-borne infections like HIV, hepatitis B virus (HBV) and hepatitis C virus (HCV). This study aims to describe the frequency and distribution of the NSIs reported in the institution over a period of four years, the factors associated with it and the immediate post-exposure prophylaxis administered.

Methods: A record-based retrospective analysis of the NSIs suffered by HCWs working in the Pradyumna Bal Memorial Hospital (PBMH), Kalinga Institute of Medical Sciences (KIMS), Bhubaneswar over a period of four years (2019 - 2022) was conducted. Data was extracted from the Exposure Prevention Information Network (EPINet) using a researcher-made proforma and analysed using Epi Info software 7.3.2.

Results: There were a total of 115 reported cases of NSIs, the incidence being 2.52%, 1.55%, 0.85% and 1.64% in the years 2019,2020,2021 and 2022 respectively. The most commonly affected age group was 18-30 years with females constituting the majority. Nurses were more commonly affected in the first two years of the study (2019 and 2020) whereas in 2021 and 2022 housekeeping staff suffered more injuries. The highest incidence of NSIs (40%-59%) was noted to have occurred in the morning shift. Inpatient department (IPD) contributed the maximum number of cases of NSIs. Out of the 93 cases with known source patient, HIV serological status was negative for all, while HBV and HCV status was positive in nine and two cases respectively. The serology status for the three viral markers (HIV, HBV and HCV) was unknown in 22 cases. After evaluation for the necessity of post-exposure prophylaxis (PEP), initial PEP was given to 14 persons for prevention of HBV and to 20 persons for HIV prevention.

Conclusion: Of the total NSIs reported over the four years, the incidence of NSIs per thousand HCWs showed a declining trend; with the younger age group and female gender reporting a higher incidence. Job category wise, housekeeping staff and nursing staff suffered more NSIs. Highest number of NSIs were reported from IPD. A greater proportion of NSIs were due to hollow bore needles. Most of the injuries occurred during daytime. Source patient could be identified in majority of the cases. HCWs who were identified to be at risk received immediate PEP as per the institutional policy. Stringent documentation of NSIs can assist in root cause analysis and to implement measures to prevent their occurrence.

## Introduction

A frequently encountered occupational hazard in healthcare settings is needle stick injury (NSI), which is defined as “any percutaneous injury, penetration of skin resulting from a needle or other sharp object, which has been in contact with blood, tissue, or other body fluids prior to the exposure”[1]. The common procedures during which NSIs may occur are injection administration, needle recapping, surgical techniques, blood collection, intravenous (IV) line insertion, suturing, blood sugar monitoring and inappropriate disposal of sharps and needles [2]. Sharp injuries are associated with transmission of blood borne pathogens like hepatitis B virus (HBV) and hepatitis C virus (HCV) and HIV [3]. The risk of transmission following percutaneous exposure is as follows - HBV: 6-30%, HCV: 1.8%, and HIV: 0.3% [4]. According to estimates from the World Health Organization (WHO), there are over three million workplace exposures to sharp injuries per year [5]. US Exposure Prevention Information Network (EPINet) recorded 16.5 injuries in 23 hospitals in 2011 for every 100 occupied beds [6]. Unfortunately, there is no national network for maintaining database and reporting of sharp injuries in India. A study conducted in Jawaharlal Institute of Postgraduate Medical Education & Research (JIPMER) reported 10.6 episodes per 100 occupied beds per year (OBY) [7]. In our hospital, we use the EPINet form for documenting needle sticks and sharp injuries. Standardized fields are available on the EPINet form for the purpose of recording, tracking, and analyzing NSIs [8].

This study aims to describe the frequency and distribution of the NSIs reported in the institution over a period of four years, the factors associated with it (namely location, employment category, injury kind, manner of injury) and immediate post-exposure prophylaxis received.

A part of the study was presented as a poster in the 45th Annual Conference of Indian Association of Medical Microbiologists held at Bhubaneswar, 2022.

## Materials and methods

Setting and data collection

A retrospective analysis of the NSI-related data of a 2600 bedded tertiary care medical college and hospital, Kalinga Institute of Medical Sciences (KIMS) and Pradyumna Bal Memorial Hospital (PBMH), Odisha in the period from January 2019 to December 2022 was conducted. This record-based study for assessing the factors related to NSIs among health care workers (HCWs) was done by collecting data using a data abstraction form. As a protocol followed by the hospital, all NSIs are reported to the Emergency Department and the information collected is stored in EPINet format. This information is stored in a standardized method at the healthcare facility and helps track the occupational exposures that put staff members at risk.

Information retrieved from this standardized format included specific information about every occurrence with a sharp device, the job category, time of injury, department of injury, kind of sharp device that caused injury and other specific information on NSIs.

Operational definitions used in the study

The term “health care worker” included all workers in the health care setting who used or were exposed to needles and other sharp devices that were contaminated with blood or other potentially infectious materials. This included doctors, nurses, technicians, housekeeping and the others group (which comprised the other 18 categories in EPINet).

Disposable syringes, hypodermic needles, phlebotomy needles, other types of needles, scalpels, scissors, razor blades and other sharp objects were included in the study's case definition of needle stick injuries.

The term " source patient" is the patient in the hospital whose blood or body fluid is present in a sharp object (as defined above) that accidentally punctures the skin of the health care worker.

Sample size and sampling technique

The sample size comprised all cases of NSIs reporting to the emergency department, during the study period, i.e. January 2019 to December 2022. During these four years a total of 115 NSIs were reported. The authors’ used a purposive sampling technique.

Data analysis

All data from EPINet forms were coded and entered in an Excel sheet (Microsoft, Redmond, WA, USA) and then imported to Epi Info software version 7.3.2. Demographic characteristics of healthcare workers, work-related factors with needle stick injuries, type of instrument causing injury and other variables were analyzed and presented in numbers and percentages. The protocol for post-exposure prophylaxis received by the HCWs was done as per standard guidelines and analyzed [9-11].

Statistical analysis

The association of various risk factors for NSIs occurring annually was analyzed and chi-square and Fisher-exact tests were used as tests of association. The incidence proportion was calculated by dividing the total number of new NSI cases over the study period by the baseline population of HCWs of that year. A p-value less than 0.05 was considered statistically significant.

Ethical considerations

Ethical permission for this study was obtained before beginning the study from the Institutional Ethics Committee, Kalinga Institute of Medical Sciences, Kalinga Institute of Industrial Technology (KIIT), Deemed to be University, Bhubaneswar (KIIT/KIMS/IEC/1054/2022). 

## Results

**Figure 1:**
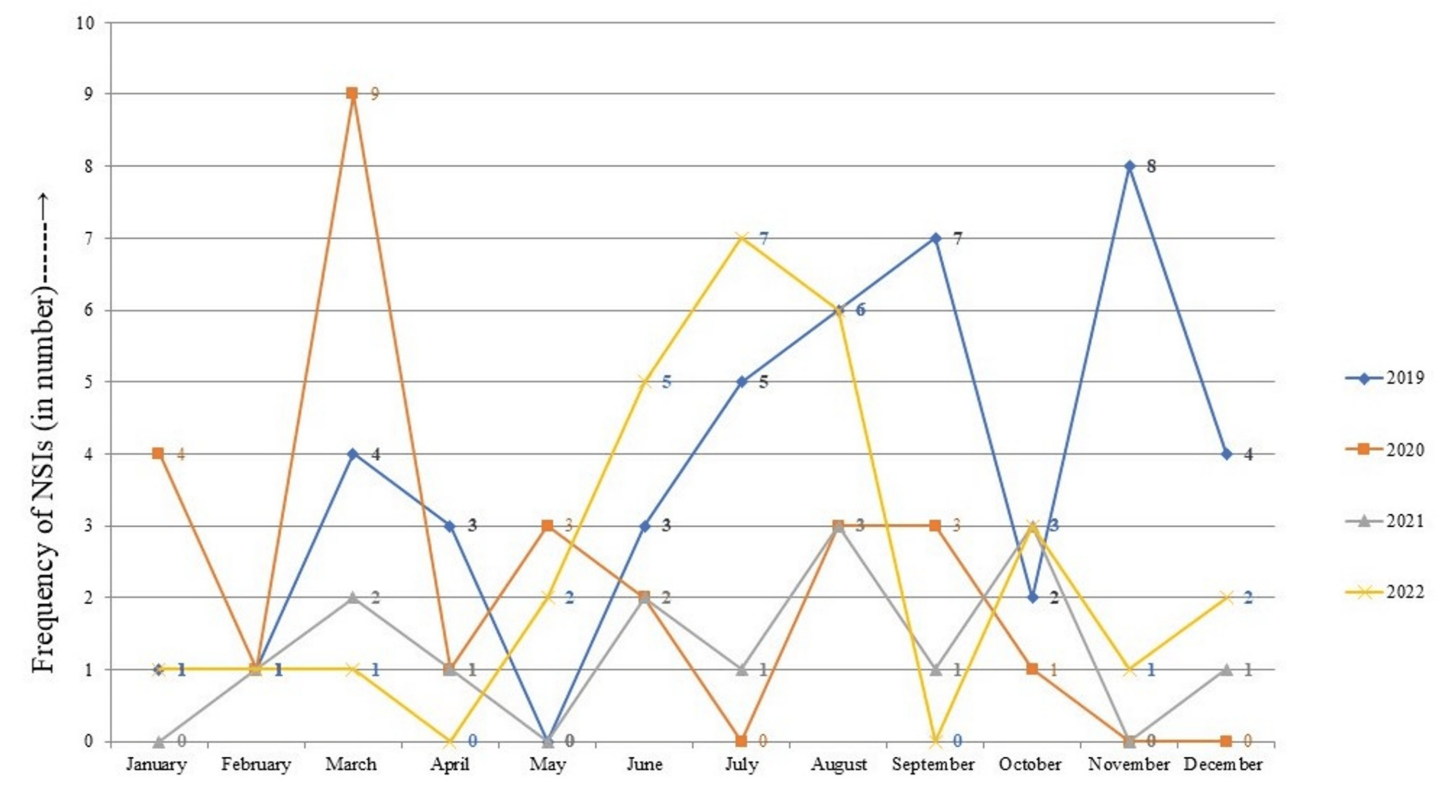
Four-year trends of needle stick injuries (NSIs) reported by health care workers (HCWs) (in numbers) The data has been represented in numbers (N)

In the four-year study period (January 2019-December 2022), there were a total of 115 reported cases of NSIs, the incidence being 2.52% (44 per 1748), 1.55%, (27 per 1744), 0.85% (15 per 1760) and 1.64% (29 per 1766) in 2019, 2020, 2021 and 2022 respectively. The month wise reporting of the NSIs with the annual trend is shown in Figure 1.

The average number of NSIs per HCW per year (being measured as rate of injury per 1000 worker per year) was calculated to be 25.17, 15.48, 8.52 and 16.42 per 1000 worker per year in the years 2019, 2020, 2021 and 2022 respectively.

NSIs were reported more in females and in the age group 18-30 years, the individual risk of housekeeping staff being 18.2%(8/44, N = 44) to 46.7 % (7/15, N= 15) (Table 1).

**Table 1:** Socio-demographic characteristics of the sampled population **rest of the 18 categories (excluding the above) as categorized in EPINet # applying fisher-exact test as test of association; ## applying chi-square test as test of association χ2= chi-square test; df=degree of freedom The frequency data has been presented as number and percentage (in brackets) NSI: Needle stick injury, HCWs: healthcare workers

Variable	2019		2020		2021		2022	
	Total HCWs (N=1748) N (%)	HCWs with NSI (n=44) N (%)	Total HCWs (n=1744) N (%)	HCWs with NSI (n=27) N(%)	Total HCWs (n=1760) N (%)	HCWs with NSI (n=15) N (%)	Total HCWs (n=1766) N (%)	HCWs with NSI (n=29) N (%)
Age group (in years)								
18-30	687 (39.30)	4 (9.09)	680 (39.00)	26 (96.30)	684 (38.86)	12 (80.00)	685 (38.78)	20 (68.96)
31-40	589 (33.70)	40 (90.91)	581 (33.31)	1 (3.70)	595 (33.81)	2 (13.33)	600 (33.98)	7 (24.14)
41-50	289 (16.53)	0 (0.00)	276 (15.83)	0 (0.00)	276 (15.68)	1 (6.67)	282 (15.97)	2 (6.90)
>50	183 (10.47)	0 (0.00)	207 (11.86)	0 (0.00)	205 (11.65)	0 (0.00)	199 (11.27)	0 (0.00)
p-value	<0.002^#^^(statistically significant)^		<0.0004^#^^(statistically significant )^		0.0001^#^^(statistically significant)^		0.0002^#^^(statistically significant)^	
	F-value=140.9; df=3		F-value=401.4; df=3		F-value1721=; df=3		F-value=649; df=3	
Gender								
Male	725 (41.48)	8 (18.18)	732 (41.97)	9 (33.33)	721 (40.97)	0 (0.00)	741 (41.96)	8 (27.59)
Female	1023 (58.52)	36 (81.82)	1012 (58.03)	18 (66.67)	1039 (59.03)	15 (100.0)	1025 (58.04)	21 (72.41)
p-value	0.0015^##^^(statistically significant)^		0.3593^##^^(statistically non-significant)^		0.0012^##^^(statistically significant)^		0.1138^##^^(statistically non-significant)^	
	χ2=10.090; df=1		χ2=0.840; df=1		χ2=10.499; df=1		χ2=2.501; df=1	
Job category of the injured worker								
Doctor	395 (22.6)	5 (11.37)	401 (23.00)	4 (14.81)	403 (22.90)	2 (13.33)	406 (22.99)	3 (10.35)
Nurse	854 (48.86)	11 (25.00)	855 (49.03)	10 (37.04)	862 (48.97)	3 (20.00)	869 (49.21)	6 (20.69)
Technician	124 (7.09)	1 (2.27)	122 (7.00)	0 (0.00)	123 (6.99)	0 (0.00)	122 (6.91)	3 (10.34)
House- keeping	185 (10.58)	8 (18.18)	192 (11.01)	9 (33.33)	193 (10.97)	7 (46.67)	195 (11.04)	9 (31.03)
Others **	190 (10.87)	19 (43.18)	174 (9.96)	4 (14.82)	179 (10.17)	3 (20.00)	174 (9.85)	8 (27.59)
p-value	<0.0001^#^^(statistically significant)^		0.0001^#^^(statistically significant)^		0.0002^#^^(statistically significant)^		<0.0001^#^^(statistically significant)^	
	F-value=1935.7; df=4		F-value=5346.6; df=4		F-value=14245.3; df=4		F-value=12339.5; df=4	

At the time of NSI, the largest proportion 40% (N= 15) to 56.82% (N=44) were using single gloves, followed by the group with no gloves (34.48 to 46.67%: N=29 and 15 respectively). Coming to the year-wise break-up: in 2019, 56.82% (25/44, N=44) were using single gloves, 2.27% (N=44) wore double gloves and 40.91% (N=44) did not wear any gloves during the occurrence of NSIs. Similarly, in 2020, 51.85% (N=27), in 2021, 40% (N=15) and in 2022,48.28% (N=29) were using single gloves, while 11.11% (N=27) in 2020, 13.33% (N=15) in 2021 and 17.24% (N=29) in 2022 used double gloves during the NSI. Those using no gloves during NSIs were 37.04% (N=27), 46.67% (N=15) and 34.48% (N=29) in 2020, 2021 and 2022 respectively.

Maximum incidence of cases around 40% (N=15) to 59% (N=27) cases occurred in the morning shift, and during use of device. The devices most commonly responsible were hollow bore needles ranging between 86.67% (N = 15) to 100% (N=29) in various years. The root cause analysis of NSI was done and found that the majority of the injuries occurred “during use of device” and “while disposing the needle” (Table 2).

**Table 2:** Details of the area, type and use of device, timing and circmstances during the NSI # rest of the 14 categories (excluding the above); ## rest of the category (excluding the above) as categorized in EPINet. The frequency data has been presented as numbers and percentage (in brackets) NSI: Needle stick injury, HCWs: healthcare workers

Variable	2019 [N=44] N (%)	2020 [N=27] N (%)	2021 [N=15] N (%)	2022[N=29] N (%)
Area of work at time of NSI/Place of NSI				
Inpatient department	20(45.46)	11(40.74)	5(33.33)	8(27.58)
Emergency/Casualty	11(25.00)	6(22.22)	1(6.66)	6(20.69)
ICU	7(15.91)	4(14.82)	4(26.67)	6(20.69)
Operation Theatre	1(2.27)	2(7.41)	1(6.67)	2(6.90)
OPD	4(9.09)	3(11.11)	0(0.00)	1(3.45)
Others#	1(2.27)	1(3.70)	4(26.67)	6(20.69)
Injured worker was using the device at time of NSI				
Yes	27(61.36)	15(55.56)	8(53.33)	25(86.21)
No	17(38.64)	12(44.44)	7(46.67)	4(13.79)
Device involved in the last accident				
Hollow bore needle	40(90.91)	21(77.78)	13(86.67)	29(100.00)
Plain bore needle	4(9.09)	6(22.22)	2(13.33)	0(0.00)
Blood on the device				
Yes	30(68.18)	15(55.56)	5 (33.33)	21(72.42)
No	1(2.27)	1(3.70)	2(13.33)	4(13.79)
Unknown	13(29.55)	11(40.74)	8(53.34)	4(13.79)
Duty shift at time of NSI				
Morning	25(56.82)	16(59.26)	6(40.00)	13(44.83)
Evening	10(22.73)	6(22.22)	5(33.33)	7(24.14)
Night	9(20.45)	5(18.52)	4(26.67)	9(31.03)
Circumstances during injury (RCA)				
During use	16(36.37)	13(48.15)	6(40.00)	11(37.93)
While recapping	10(22.73)	2(7.41)	3(20.00)	6(20.69)
Device left on floor	1(2.27)	2(7.41)	1(6.67)	0(0.00)
While cleaning	0(0.00)	0(0.00)	0(0.00)	1(3.45)
While disposing	15(34.09)	6(22.22)	4(26.66)	7(24.13)
After disposal	1(2.27)	0(0.00)	1(6.67)	2(6.90)
Others##	1(2.27)	4(14.81)	0(0.00)	2(6.90)

Superficial injuries were predominant ranging from 79% (N=29) to 93% (N=15) in various years and most of the HCWs received medical attention (93% to 100%) immediately. Around 93% (N=44) to 100% (N=29) washed their hands with soap and water and 37% (N=27) to 65% (N=29) of the affected HCWs reported to the nodal center within 30 minutes of exposure (Table 3).

**Table 3:** Experience after the Needle Stick Injury The frequency data has been presented as number and percentage (in brackets)

Variable	2019 [N=44] N (%)	2020 [N=27] N (%)	2021[N=15] N (%)	2022 [N=29] N (%)
Injury type				
Superficial (little or no bleeding)	37(84.09)	22(81.48)	14(93.33)	23(79.31)
Moderate (skin punctured, some bleeding	7(15.91)	5(18.52)	1(6.67)	5(17.24)
Severe (deep stick/cut, or profuse bleeding)	-	-	-	1(3.45)
Receive medical attention within 2 h after injury				
Yes	41(93.18)	27(100.00)	14(93.33)	29(100.00)
No	3(6.82)	0(0.00)	1(6.67)	0(0.00)
Action taken after injury				
Washed with soap and water	41(93.18)	27(100.00)	14(93.33)	29(100.00)
Got tested for HIV, hepatitis B, and hepatitis C	44(100.00)	27(100.00)	15(100.00)	29(100.00)
Time interval between NSI & reporting				
<30 minutes	26(59.09)	10(37.04)	8(53.33)	19(65.52)
30 mins- 2 hrs	13(29.55)	10(37.04)	1(6.67)	5(17.24)
2-12 hours	3(6.82)	7(25.92)	2(13.33)	5(17.24)
12-24 hours	1(2.27)	-	4(26.67)	-
>24 hours	1(2.27)	-	-	-

After the NSI, the serological workup for viral markers (HBV, HIV and HCV) of both the HCW and the source patient (if identifiable) was conducted. All the affected HCWs tested negative for the viral markers.

**Figure 2:**
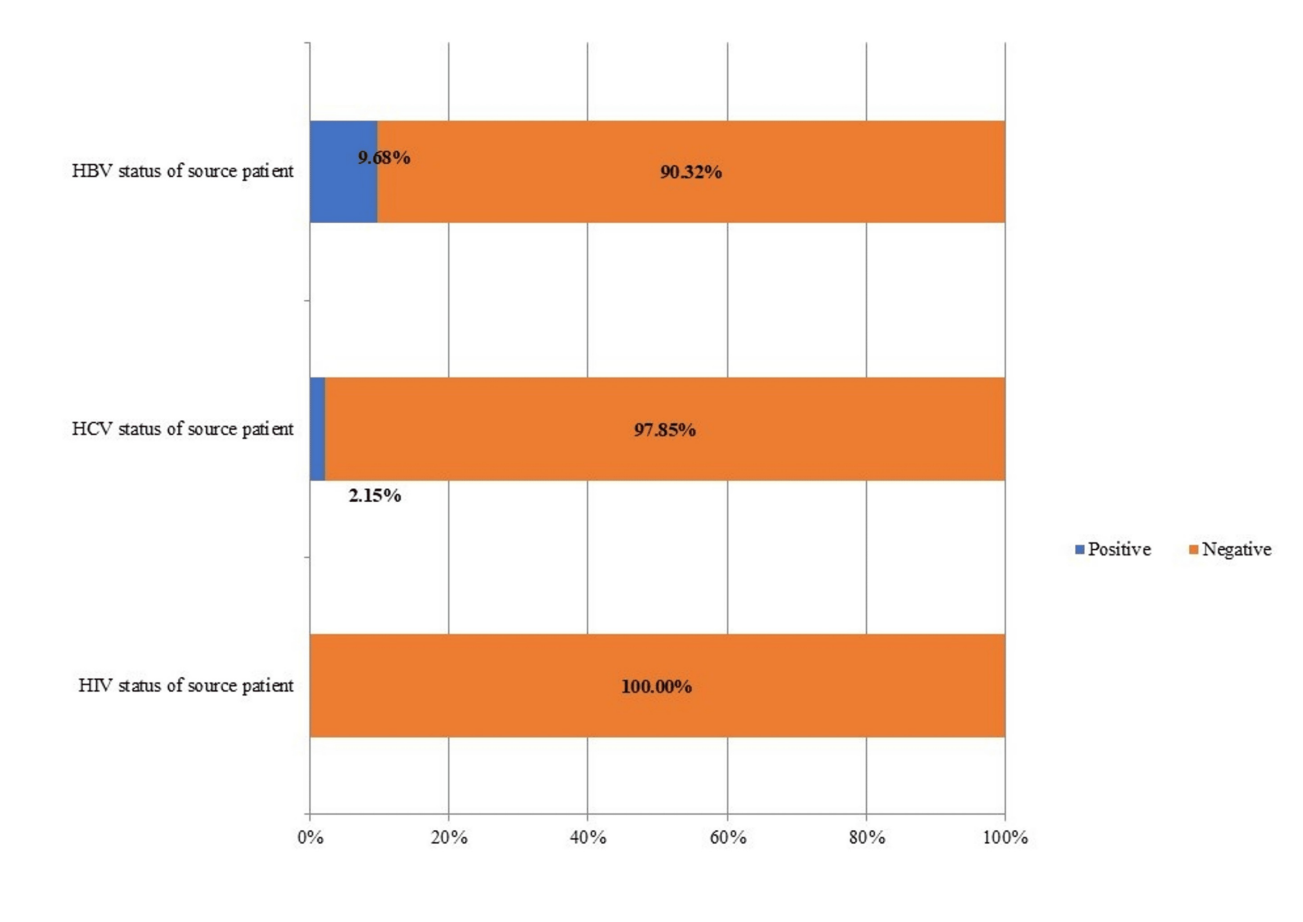
Serological Status of the known source patient for blood-borne viruses at time of NSI in percentage [N=93] The data of the serological status has been presented as percentage NSI: Needle stick injury, HBV: hepatitis B virus, HCV: hepatitis C virus

The source patient was identifiable in 93 cases (80.8%, N=115) of NSIs in which, HIV serological status was negative in all the cases. HBV and HCV status was positive in nine and two cases respectively (Figure 2).

Of the nine HCWs who were exposed to HBV-positive sources, six had protective hepatitis B surface antibody titres. Hence no further action was taken for them. PEP for HBV was indicated for the rest three HCWs whose antibody titres were not protective. So, they were given first dose of hepatitis B immunoglobulin along with the first dose of HBV vaccine and were asked to complete the full course of vaccination. Two HCWs who got NSI from HCV-positive patients were counselled as per the hospital protocol.

Twenty-two HCWs (19.1%, N=115) suffered NSIs from unknown source and were assumed to have got the injury from a positive HIV, HBV and HCV source. As per protocol (after determining the exposure code and source status code) 10 of the 22 HCWs were eligible for PEP and were provided with the first dose of PEP for HIV and were referred to antiretroviral therapy (ART) centre for further evaluation. For HBV, protective hepatitis B surface antibody titers were found in 11 (out of 22) of them and hence no further action was taken. The rest 11 were given PEP for HBV. Hence a total of 14 affected persons (three source known and 11 source unknown), were given PEP for HBV. For HCV, no immediate action was taken for 22 HCWs who had the NSI from unknown source.

## Discussion

One of the important professional hazards to healthcare workers is NSIs sustained while administering healthcare. Reuse of syringes, recapping of needles and unsafe sharps waste management are the main factors leading to needle stick injuries. Following exposure, the risk of infection to healthcare workers depends on various factors, prominent among them being the type of needle used, depth of injury, volume of blood involved in exposure, viral load present in the blood/body fluid at the time of exposure, delay in performing first aid and delay in starting appropriate PEP.

In this retrospective study which analyses the NSIs among HCWs over a period of four years (2019-2022), a total of 115 NSIs occurred, the incidence per thousand HCW being 2.5, 1.5, 0.8 and 1.6 in 2019, 2020, 2021 and 2022 respectively. As per WHO, the average number of NSIs per HCW varied from 0.2 to 4.7 per year which is quite similar to our finding [12].

The incidence of needle stick injury events showed a steady decline from 2019-21, with an increase again in 2022. This may be due laxity in adherence to infection control practices when the pandemic was receding or it maybe due to lack of knowledge among the newly joined staff in the post-COVID phase.

The most common age group affected in our study was 18-30 years (54%, N=115) which was in concordance with the findings of studies done in Jordan and India, where the most common age group affected with NSIs was 20-30 years [7,13].

Majority of the HCWs who reported NSIs were females, with the prevalence among females being 81% (N=44), 66.6% (N=27), 100% (N=15) and 70% (N=29) in 2019, 2020, 2021 and 2022 respectively. Overall, the female :male ratio was 3.6:1. Similar gender distribution was seen in two other studies [7,14]. But there are other Indian and international studies where male preponderance has been seen [11,15]. The increased incidence of NSI among females in our study could be attributed to the increased number of females in the workforce of our hospital.

Nursing staff were predominantly affected in 2019 and 2020, whereas in 2021 and 2022 housekeeping staff suffered more NSIs. A study in Saudi Arabia reported that housekeeping staff were mostly affected, but most of the studies have reported more injuries among the nursing staff [13,16,17]. Not using proper personal protective equipment during handling and disposal of needles combined with inadequate awareness and knowledge among housekeeping staff could have contributed to increased cases of NSIs.

In the study period from 2019-2022, 34.48% (N=29) to 46.67% (N=15) were not wearing any gloves during the NSI, while 40% (N=15) to 56.82% (N=44) were wearing gloves, in contrast to a study in Saudi Arabia where 16.6% were wearing no gloves and 75.7% were wearing gloves [18]. An Indian study by Sriram et al. showed that 65% of HCWs were wearing gloves at the time of the incident [19]. Various reasons can be ascertained starting from unavailability of gloves, lack of proper awareness on use of gloves and the workload being high. Being a retrospective record-based study, the reasons for not using gloves could not be ascertained. The authors recommend greater in-depth interviews amongst the staff sustaining NSIs, for identification of the cause.

Inpatient department reported the highest number of NSIs (38.2%, N=115) followed by casualty (21.8%, N=115) and ICU (18.2%, N=115), which is accordance with a study by Sastry et al. and Saadeh et al. but in contrast to a study conducted in a hospital in North India [7,13,15]. 

As per our findings, NSIs most commonly occurred during use of the device i.e. 36.4% (N=44) to 48.1% (N=27) which is similar to the findings of Alsabaani et al. but in contrast to the observation of Lal et al., who found that recapping was the commonest cause of NSI [14,20]. In our hospital as part of the training programme on standard precautions we discourage needle recapping. Although the exact patient care activity that led to NSI was not analyzed in this study, various other patient care activities like drawing blood, inserting needles, administering injections and even restraining a violent patient may have led to the injuries.

Hollow bore needles were involved in NSIs in 65.3% and 81% of cases in a study in Qatar and Malaysia respectively whereas a greater proportion (86.67% to 100.00%) of NSIs in our study could be attributed to hollow bore needles [21,22]. It is noteworthy that hollow bore needles are more efficient in transmitting blood borne viruses than solid needles as they carry higher quantity of blood [23]. Additionally, a study has observed that the presence of visible blood on a device carries a greater risk of acquiring infection [24]. In our study, in around 61.7% of NSIs, blood was visible on the device.

Majority of the injuries occurred during the day shift followed by evening shift which was similar to the findings of Jahangiri et al. [23]. The increased workload during the day shift and the diversity of tasks that the healthcare workers have to perform may be a cause for increase in risk of NSIs.

Most of the injuries sustained were superficial (79% to 93% in the four years) in nature in our study. In a study done in Ethiopia, the majority of injuries were moderate in nature, whereas in an Indian study the superficial injuries were more in number [15,17].

Majority (93% to 100%) of the NSIs were reported to the nodal centre and received medical attention within two hours of injury which indicates that our infection control practices are stringent and implementation of the guidelines are satisfactory. The compliance to first aid protocol measures like washing hands with soap and water is quite high in our study (93% to 100% ), which is in contrast to low compliance seen in a study done in Iran [24].

The source patient was identifiable and the status of viral markers (HIV, HBV and HCV) could be known in around 80.8% (93/115) of NSI cases which is higher than the data reported by Alsabaani et al. where source patients could be identified in only 35.8% cases. This could be probably because our study is done in a single centre, whereas the study by Alsabaani et al. included all levels of healthcare [14]. Goel et al. reported that the source was known in around 91.4% cases which is higher than our finding. This can be explained by the fact that their study was a prospective study and hence the follow-up could be better done than our study which is retrospective in nature [15]. Of the total 115 HCWs who sustained NSIs, 14 (12.1%) affected persons were given immediate post-exposure prophylaxis for HBV and 20 (17.3%) persons were given first dose of PEP for HIV in our hospital. Hence a total of 34 (29.5%, N=115) healthcare workers received first dose of PEP which concurs with the findings of Sastry et al. where 30.5% of HCWs received first dose of PEP [7].

Limitations of the study

This is a single-centre retrospective study using purposive sampling technique, which has analyzed the trend of NSIs over a period of four years. Being retrospective in nature, we may have missed some undocumented data which has not been documented. Inaccuracies may also have crept in during documentation. Being a single-centre study from a tertiary care National Accreditation Board for Hospitals and Healthcare Provider (NABH)-accredited hospital, where there is strict adherence to infection control practices, the study results may not be generalisable to the NSI incidence and risk factors at large. This study would have given us more in-depth information if it had involved more healthcare centers.

Recommendations from the study

The findings of this study helped guide the development of educational and awareness programs targeted to the identified specific risk categories, so that such injuries could be effectively prevented in future. Further research can be done among these group of HCWs to assess their knowledge, attitude and practice regarding NSIs following their training and for checking the effectiveness of the tailored planned intervention program.

## Conclusions

NSIs still constitute a major occupational hazard for healthcare professionals. Strict adherence to safe injection practices, proper disposal of sharps, continuous training activities to increase awareness among the HCWs are some of the key strategies for reducing NSIs. The findings of this study helped us identify bottlenecks for prevention of NSIs and further research has been planned to determine the efficacy of a planned targeted intervention to decrease NSIs, as it is largely preventable.
